# The Treatment of Scalp Wounds

**Published:** 1907-05-25

**Authors:** H. Drinkwater


### THE TREATMENT OF SCALP WOUNDS.

By H. DRINKWATER, M.D.

For the past ten years or thereabouts I have adopted a plan of treatment of wounds of the scalp and face that has proved very successful—far more satisfactory in every respect than my previous practice, which was that commonly in vogue and still used by most medical men with whom I have discussed the subject.

I see a great number of cases of injury to the head and face amongst the men employed at a large colliery in North Wales.

The plan adopted has several advantages—it is simple, inexpensive, and time-saving—and the time-saving is an advantage both to myself and the patient. The wounds heal more quickly and almost invariably by "first intention," so that the patient is able to return to his work earlier than was formerly the case. However extensive the wound, one may expect rapid and complete healing without suppuration.

The plan is what may be called the dry-exposed method, and consists in (1) thorough cleansing and washing with biniodide solution; (2) accurate apposition by sutures; (3) dusting with powdered boracic acid; (4) giving the patient a supply of boracic acid which is to be dusted over the wound often enough to keep the surface dry. Whenever there is the least moisture (blood or lymph) visible the powder is to be applied. This generally means every few hours for the first day, and after that two or three times a day. No dressing whatever is applied, the air has free access to the surface of the wounded part, and evaporation helps to keep the part dry. There is no culture fluid for germs to grow in.

I find the subsequent cicatrix smaller than in wounds that are kept covered up. The sutures are removed on the sixth or seventh day.

If there is free arterial bleeding a pad and bandage is applied (after suturing the wound) and left on for one day only.

